# Hemin induces autophagy in a leukemic erythroblast cell line through the LRP1 receptor

**DOI:** 10.1042/BSR20181156

**Published:** 2019-01-03

**Authors:** Ruben Adrian Grosso, Paula Virginia Subirada Caldarone, María Cecilia Sánchez, Gustavo Alberto Chiabrando, María Isabel Colombo, Claudio Marcelo Fader

**Affiliations:** 1 Universidad Nacional de Cuyo, Facultad de Odontología, Mendoza, Argentina; 2 Consejo Nacional de Investigaciones Científicas y Técnicas (CONICET), Instituto de Histología y Embriología (IHEM), Facultad de Ciencias Médicas, Universidad Nacional de Cuyo, Mendoza, Argentina; 3 Centro de Investigaciones en Bioquímica Clínica e Inmunología (CIBICI), Consejo Nacional de Investigaciones Científicas y Técnicas (CONICET), Departamento de Bioquímica Clínica, Facultad de Ciencias Químicas, Universidad Nacional de Córdoba, Córdoba 5016, Argentina

**Keywords:** autophagy, cell differentiation, LRP1, trafficking

## Abstract

Hemin is an erythropoietic inductor capable of inducing autophagy in erythroid-like cell lines. Low-density lipoprotein receptor-related protein 1 (LRP1) is a transmembrane receptor involved in a wide range of cellular processes, such as proliferation, differentiation, and metabolism. Our aim was to evaluate whether LRP1 is responsible for hemin activity in K562 cells, with the results demonstrating a three-fold increase in LRP1 gene expression levels (*P*-values <0.001) when assessed by quantitative real-time RT-PCR (qRT-PCR). Moreover, a 70% higher protein amount was observed compared with control condition (*P*-values <0.01) by Western blot (WB). Time kinetic assays demonstrated a peak in light chain 3 (LC3) II (LC3II) levels after 8 h of hemin stimulation and the localization of LRP1 in the autophagosome structures. Silencing LRP1 by siRNA decreased drastically the hemin-induced autophagy activity by almost 80% compared with control cells (*P*-values <0.01). Confocal localization and biochemical analysis indicated a significant redistribution of LRP1 from early endosomes and recycling compartments to late endosomes and autophagolysosomes, where the receptor is degraded. We conclude that LRP1 is responsible for hemin-induced autophagy activity in the erythroblastic cell line and that hemin–LRP1 complex activation promotes a self-regulation of the receptor. Our results suggest that hemin, via the LRP1 receptor, favors erythroid maturation by inducing an autophagic response, making it a possible therapeutic candidate to help in the treatment of hematological disorders.

## Introduction

Erythroid maturation is a highly regulated process where immature cells from bone marrow pass through a series of differentiation stages in order to become mature red blood cells [1,2]. During this process, essential intracellular modifications take place such as nuclear extrusion, hemoglobin synthesis, protein membrane regulation, and the degradation of entire organelles that are unnecessary for erythrocyte functionality [3,4].

Autophagy is a lysosomal degradative pathway, in which macromolecules and entire organelles are surrounded by double-membrane vesicles called autophagosomes and then targetted to lysosomes for their degradation [5–7]. Autophagy participates actively in erythropoiesis, being responsible for engulfing and eliminating the mitochondria (mitophagy) and ribosomes once all the hemoglobin has been synthesized [8,9]. Since autophagy is known to be implicated in diverse cellular processes, numerous kinds of autophagic inductors have been studied, such as starvation medium, rapamycin, and resveratrol [10,11]. Hemin, an erythropoiesis inductor, is capable of stimulating erythroid cell maturation and promoting hemoglobin synthesis [12,13]. Moreover, this compound is able to generate an autophagic response in erythroleukemia cells lines, thereby inducing the expression of some autophagic genes including light chain 3 (*LC3*), autophagy related (Atg) 5 (*Atg5*), and *Beclin1* [14]. Furthermore, it has been demonstrated by biochemical and ultrastructural approaches that hemin stimulates mitophagy in the chronic leukemia K562 cell line, which also requires the NIX protein active form [14].

Low-density lipoprotein receptor-related protein 1 (LRP1) is a transmembrane endocytic receptor associated with more than 40 extracellular ligands. Structurally, LRP1 is composed by an extracellular α-chain with four ligand-binding cysteine-rich domains, as well as one *β*-chain with an intracytoplasmic domain that participates in activating different signaling pathways [15]. Ligand–receptor complex triggers clathrin-dependent endocytosis and initiates a signaling cascade that activates different proteins downstream, such as phosphatidylinositol-4,5-bisphosphate 3-kinase (PI3K)/protein kinase B (AKT), extracellular signal-regulated protein kinases 1 and 2 (ERK1/2), and mitogen-activated protein kinase (MAPK) [16–19]. This receptor is involved in many cellular processes including the molecular metabolism of α-2-macroglobulin and apolipoprotein E, cell migration, apoptosis, and autophagy [20–22]. Concerning trafficking, once internalized, LRP1 accumulates in early endosomes and releases the ligand to late endosomes. In addition, LRP1 commonly colocalizes with the early endosomal marker, early endosome antigen 1 (EEA1) [20,23–25], and then the receptor continues the recycling pathway through vesicles containing the Rab11 and membrane-type matrix metalloproteinase 1 (MT1-MMP) proteins before its translocation to the plasma membrane in retinal cells [25]. LRP1 has also been found to localize in recycling compartments marked with the sorting nexin 17 (SNX17) protein, before reaching the basolateral cellular membrane in neurones [26]. However, this receptor has not been frequently found in later endosomal compartments once the LRP1–ligand complex has been released [20].

LRP1 participates as a scavenger receptor for the hemin–hemopexin complex in hepatic cells [27], leading to its endocytic internalization and metabolism, but to date no information is available about LRP1 function in erythroid cells. Recently it was demonstrated that LRP1 is present in K562 cells at low levels compared with other cancer cell lines and this regulation might be implicated with either a better or worse prognosis of malignancy [28]. Furthermore, in placenta cells, LRP1 is involved in regulating intracellular iron levels in association with the cellular receptor of the feline leukemia virus subgroup C (FLVCR1), an exporter of the heme group to the extracellular matrix [29,30]. Likewise, platelet factor 4 (PF-4), a negative regulator of megakaryopoiesis, is related to LRP1, with this receptor being expressed transiently during this process but being absent from mature platelets. Silencing of LRP1 with shRNA blocks the effect of PF4 and leads to normal megakaryocytic maturation [31].

These above findings led us to speculate that LRP1 may be involved in the hemin-stimulated autophagy pathway. In the present study, we demonstrated that induction of leukemia K562 cells with hemin, not only enhanced the autophagy pathway, but also activated overexpression of the LRP1 protein and gene. Interestingly, we demonstrated that hemin targets LRP1 to autophagosomes, with this receptor being in part responsible for hemin autophagy activation, due to the fact that silencing of LRP1 decreased the lipidated form of the LC3 protein. Likewise, hemin induced a traffic modification of LRP1, thereby increasing its localization in the later endosomal compartments, including the lysosomal vesicles, for its subsequent degradation.

## Materials and methods

### Cell culture and stimulation solutions

Erythroblastic cell lines (K562 and HEL 92.1.7) and HeLa cell lines were cultured in RPMI 1640 and D-MEM medium respectively with 10% FBS at 37°C and 5% CO_2_, with stimuli being added after replacing the complete medium. Hemin Chloride (Calbiochem) was prepared with sodium hydroxide 0.2 M as a diluent and was used at final concentration of 50 µM. Bafilomycin A1 (Sigma–Aldrich) was prepared in DMSO and diluted in culture medium at a 100 nM. Resveratrol (Sigma–Aldrich) was diluted in Ethanol 70% and used at 100 µM stimulation concentration. Erythropoietin (EPO) (Sigma–Aldrich) was added at a final concentration of 0.2 UN. Lysosomal marker Lysotracker Red (Thermo Fisher Scientific) was incubated for 1 h. For protein synthesis inhibition, 10 µM of cycloheximide (Sigma) was used for 4 h.

### Plasmid transfection

GFP-Rab5 wild-type, GFP-CD63 wild-type, GFP-Rab7 wild-type, RFP-Cathepsin D wild-type, SP-HA-GFP-mLRP4 (composed of the LRP1 signal peptide (SP), hemagglutinin (HA) epitope, and GFP in their N-termini, followed by the fourth ligand binding, and transmembrane and cytoplasmic domains of LRP1) and siRNA-LRP1 plasmids were used. A total of 5 × 10^6^ cells was used for each transient transfection by electroporation, using standardized protocols. For siRNA-LRP1, we used Silencer® Select pre-designed siRNA for the LRP1 (Ambion) sequence 5′–3′: GCUGGUUGCUUGUACAACAtt (sense), UGUUGUACAAGCAACCAGCtg (antisense) and performed a double-hit electroporation with an interval of 24 h between each hit.

### Antibodies

For Western blot (WB) and immnufluorescence, we employed: rabbit anti-LRP1β (Sigma–Aldrich Canada), rabbit anti-LC3B (Sigma–Aldrich), mouse anti-β-actin (GenScript), mouse anti-GM130 (Sigma), anti-rabbit HRP (Sigma), mouse anti-HRP (Sigma), Alexa Flour 488 anti-rabbit (Sigma), Cy3 anti-rabbit (Sigma), Cy3 anti-mouse, and Alexa Flour 594 anti-rabbit (Sigma).

### SDS/PAGE and WB

After stimulation, cells were washed with PBS 1× and then lysed with a solution containing Triton X-100, protease and phosphatase inhibitors. The total protein was quantitated by Bradford. For WB, we used 100 µg of total protein on 10–15% of SDS/PAGE, which was then transferred to a nitrocellulose membrane. Blocking was performed for 1 h with 5% fat-free milk in a 0.1% Tween 20 PBS solution. After two washes with PBS 1×, the primary antibodies and peroxidase–conjugated secondary antibodies were incubated. Images were obtained using the chemiluminescent ImageQuant LAS 4000 camera system, and analyzed in ImageJ Software.

### Indirect immunofluorescence

For cell fixation, we used 4% paraformaldehyde for 30 min. Autofluorescence was blocked for 30 min with 50 mM NH_4_Cl in PBS, and cells were permeabilized for 20 min with 0.05% saponin in PBS containing 0.2% BSA. Primary antibodies were incubated for 2 h and secondary antibodies for 1 h at room temperature. Antibodies were prepared in PBS solution containing 1% BSA. Coverslips were mounted on glass slides, and examined with an Olympus FV-1000 fluorescence confocal microscopy. Images were analyzed by ImageJ software, due to the fact that K562 cells are quite small (12–18 μm).

### Trypan Blue cell viability assay

K562 cells were incubated with hemin [50 µM] for 8, 24, 48, and 72 h and also without hemin stimulation (Ctl). After incubation, cells were centrifuged and washed with PBS 1× at 37°C to remove all the medium. Trypan Blue was added following the manufacturer’s guidelines. The percentage of cell death was obtained by analyzing the number of Trypan Blue dyed cells by the total cell number, counted (*n*=1000) in triplicate for each independent assay (*n*=3).

### Flow cytometry

K562 cells were incubated in six-well plates and treated with hemin [50 µM]. Then, 10^6^ cells were harvested for each group, which were washed with PBS and fixed with 2% PAF. Monoclonal anti-LRP1 at a 1/100 dilution was incubated for 1 h and then with a secondary FITC-anti-rabbit for 40 min. An analysis was performed using a FACSAria III cell sorter (BD) emitting an excitation laser light at 488 nm and counting 3 × 10^4^ events for each condition. Fluorescence was measured at 488 nm to obtain the amount of FITC fluorescence. Data were analyzed by using FlowJo software (BD).

### Semi-quantitative real-time RT-PCR

RNA from K562 cells incubated in complete medium or under hemin and resveratrol stimulation was isolated with TRIzol reagent according to protocols provided by the manufacturer (Life Technologies). The cDNA was synthesized from 4 μg of purified total RNA using a commercial kit, after which 200 nM of human LRP1 primer were used for PCR amplification. Relative expression was measured using Applied Biosystems 7500 Real-Time PCR Systems. The values were normalized against cyclophilin and analyzed by Applied Biosystem 7500 Sequence Detection Software v.1.4.

### Statistical analysis

A Relative Optic Densitometric analysis on the immunoblots was carried out using ImageJ software. The *p*-values for the densitometric analysis for the comparison of vesicular colocalization were determined by ANOVA and the *Student’s t test* using GraphPad Prism software. For multiple comparisons we used *Tukey* and *Dunnett* (compare with the control group). Descriptive and statistical significance analysis was performed by GraphPad Prism.

## Results

### Hemin induces LRP1 gene expression and protein synthesis in K562 cells

**Figure 1:**
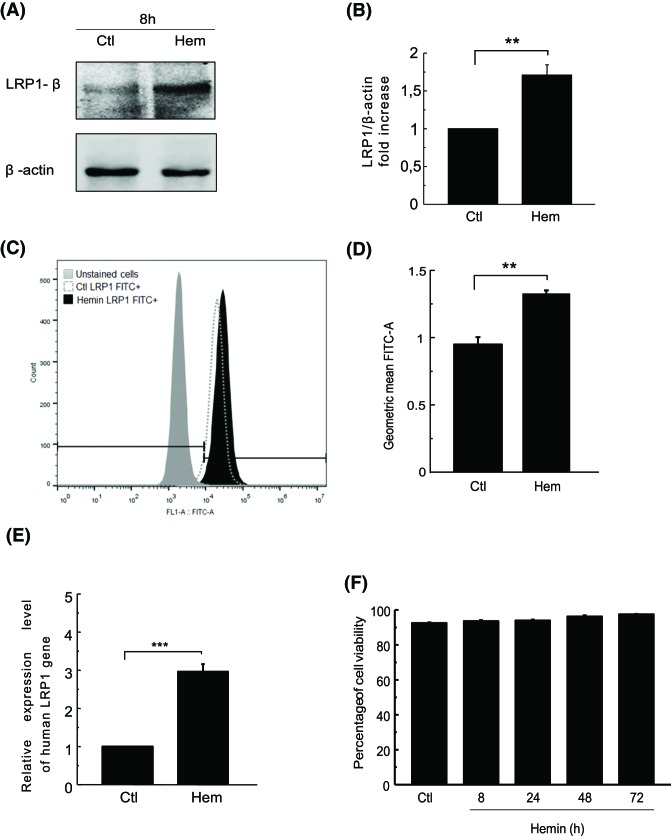
Hemin induces LRP1 gene expression and protein synthesis in K562 cells K562 cells were incubated in complete medium (Ctl) or hemin [50 µM] (hem). (**A**) After 8 h of stimulation, K562 cell lysate was subjected to WB. For protein detection, specific primary antibodies were incubated and then secondary antibodies containing HRP were also incubated. *β*-actin was used as the loading control. (**B**) Quantitation of relative optic density (ROD) was measured with ImageJ software. (**C**) Flow cytometry analysis: measurement of the FITC area on a logarithmic scale of stimulated and non-stimulated cells compared with unstained cells (negative isotype). (**D**) Graph of quantitation of geometric mean of FITC-A by Flow Jo software. (**E**) After 8 h of the above-mentioned incubation quantitative real-time RT-PCR (qRT-PCR) was performed with RNA obtained from the TRIzol method and human LRP1 [200 nM] primer. Curve analysis and quantitation of relative expression were performed with Real Time Biosystem software. (**F**) Quantitation of cell viability of non-stimulated (Ctl) and hemin-stimulated K562 at different times. Data represent mean ± S.E.M. of three independent experiments. Statistical *t*test and Dunnett’s test were also performed. The significance of the *p*-values corresponds to *p*<0.01 (**), and *p*<0.001 (***).

We have previously demonstrated that hemin is able to induce a partial maturation response, which activates autophagy/mitophagy in the K562 cell [14]. As hemin has been described as a LRP1 ligand, we analyzed whether hemin was able to modify the LRP1 receptor levels in leukemia cells during erythroid maturation. To carry this out, an SDS/PAGE immunoblot was made of K562 cells incubated for 8 h in the absence of stimulation (Ctl) and with hemin (Figure 1A). LRP1 intracellular domain (LRP1*β*) was detected by WB and compared with *β*-actin protein (Figure 1A).

After 8 h of stimulation with hemin, the K562 cells showed a significant increase in LRP1 protein levels compared with control cells, with a quantitation of the total receptor level revealing that hemin promoted an arise of ∼70% (Figure 1B). To corroborate this increase in the amount of LRP1, K562 cells were incubated in the presence of hemin and LRP1 was measured by flow cytometry (Figure 1C,D). Moreover, to determine whether this stimulus could promote not only an increase in protein levels, but also an enhancement of the transcription of *LRP1* gene, reverse transcription-quantitative PCR (RT-qPCR) was performed in K562 cells incubated under the same conditions as those mentioned above. Interestingly, quantitation by real-time software and statistical analysis of these results demonstrated that hemin increased the relative expression of LRP1 (three-fold) in hemin stimulated cells (Figure 1E). These results therefore suggest that hemin was able to induce mRNA transcription of LRP1 and thereby enhance the protein amount in K562 cells.

To evaluate whether hemin was affecting the maintenance of cell integrity, we performed a cell viability assay with Trypan Blue in response to hemin for up to 72 h of stimulation, and observed that cell viability was ∼93% in the control condition and still stable 72 h after hemin incubation (Figure 1F). Taken together, these results demonstrate that hemin induces the transcription of LRP1, which leads to LRP1 protein synthesis in K562 cells without affecting cell integrity.

### Hemin induces the colocalization of LRP1 and LC3 in a time-dependent manner

**Figure 2:**
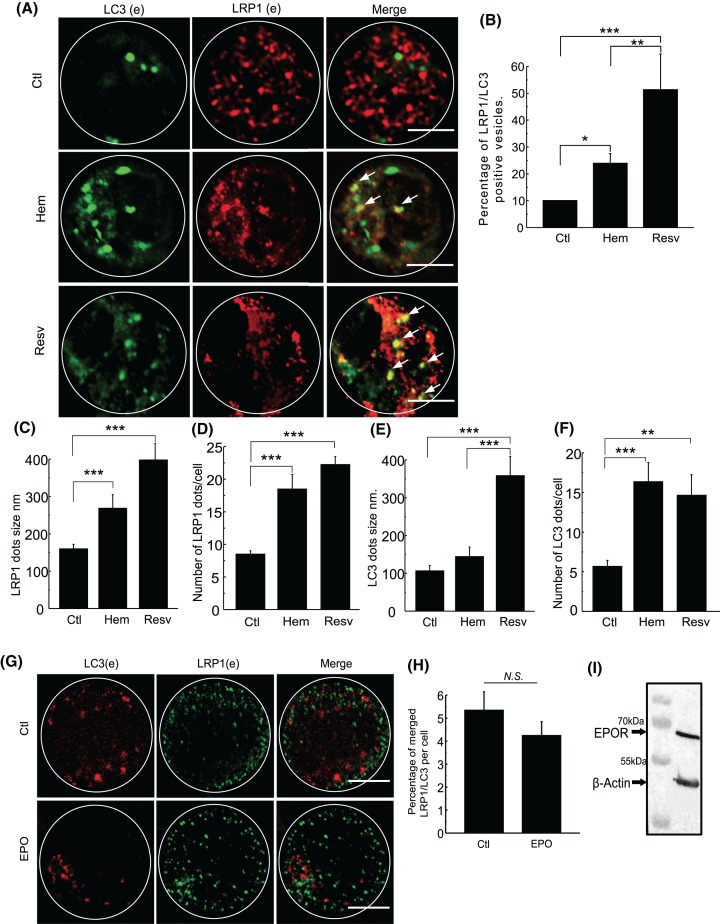
Hemin produces localization of LRP1 in the autophagy pathway K562 cells were incubated with the different stimuli hemin [50 µM] (hem), resveratrol [100 µM] (resv), and EPO [0.2 UN] for 24 h and compared with non-stimulated cells (Ctll). (**A**) Indirect immunofluorescence (IF) of K562 cells in control, hemin, and resveratrol conditions. Endogenous proteins were tagged with the primary antibodies anti-LRP1 mouse (1:600), anti-LC3B rabbit (1:400), and with the following secondary antibodies coupled with fluorescent protein: anti-rabbit Alexa Fluor 488 (1:300) and anti-mouse Cy3 (1:300). Confocal images were obtained with Olympus Fluorescent Confocal microscopy. White arrows indicate colocalizing vesicles. Scale bar = 5 µm. (**B**) Quantitation of percentage of merged LRP1/LC3 vesicles per cell using ImageJ Colocalization Finder software. (**C**–**F**) Quantitation of amount and size of LRP1 and LC3 vesicles with ImageJ analyze particles software. (**G**) Confocal images of IF of K562 cells in control and EPO conditions. Endogenous LC3 and LRP1*β* were tagged with primary and secondary antibodies coupled with anti-Rabbit Cy3 and anti-Mouse Alexa Fluor 488, respectively. Scale bar = 5 µm. (**H**) Quantitation of percentage of merged LRP1/LC3 vesicles per cell with ImageJ Colocalization Finder software. Data represent mean ± S.E.M. of three independent experiments. Forty cells for each experiment were analyzed. (**I**) WB of K562 cell to detect EPO receptor (EPOR) with anti-human EPOR (1:1000), *β*-actin was used as the loading control. Pearson values were compared statistically. Statistical *t* test was performed. The significance of the *p*-values corresponds to *p*<0.05 (*), *p*<0.01 (**), and *p*<0.001 (***).

As mentioned above, we have previously demonstrated that hemin enhances autophagy in K562 cells [14]. As it has been shown that hemin is a ligand of LRP1 we decided to study the possible role of this receptor in the autophagy pathway. To address whether the increased amount of LRP1 in cells incubated in the presence of hemin was associated with a rise in the number of autophagosomes, K562 cells were incubated in the absence (Ctl) or presence of hemin (Hem) or resveratrol (Resv) for 24 h, with the latter being added to determine whether another autophagy inductor could stimulate LRP1 in the same manner. After being fixed cells were stained with antibodies against the endogenous protein LC3 and LRP1*β*. As shown in Figure 2A,B, an increased percentage of LRP1 and LC3 positive structures was observed in hemin and resveratrol induced cells, which was 150 and 400% higher, respectively, than the control. In addition, we observed a significant rise in the number and the size of LRP1 or LC3 vesicles in cells incubated in the presence of hemin or resveratrol (Figure 2C–F).

Since EPO is also an erythropoietic inductor, we evaluated whether K562 could be stimulated by EPO in the same manner as hemin, by localizing LRP1 in the autophagosomes. IF was performed in non-stimulated (Ctl) and stimulated K562 cells with EPO for 24 h. After being fixed, we incubated the cells with antibodies against LC3 (e) and LRP1β (e) (Figure 2G), and a colocalization analysis demonstrated that EPO was not able to induce an increase in the number of LRP1 or LC3 positive structures (Figure 2H). Moreover, EPO was not capable of inducing an increment in the amount of LRP1 positive vesicles (data not shown). In order to demonstrate that EPO receptor was present in this cell line, the total amount of this receptor was analyzed by WB in K562 cells (Figure 2I), indicating that hemin increased the amount of LRP1 vesicles and improved its mobilization to autophagosomes, with these events not being mediated by other erythroid maturation inductors such as EPO.

**Figure 3:**
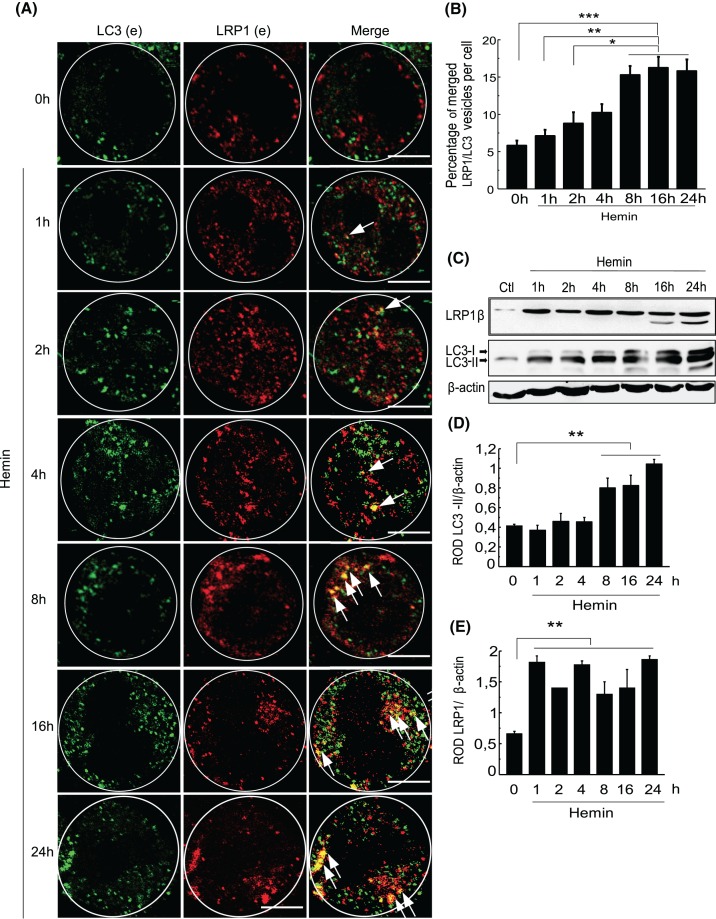
Hemin induces LRP1 and autophagy in a time-dependent manner K562 cells were stimulated with hemin [50 µM] at different times: 1, 2 4, 8, 16, and 24 h. (**A**) Indirect immunofluorescence of K562 cells in control, hemin, and resveratrol conditions. Endogenous proteins were tagged with the primary antibodies anti-LRP1 mouse (1:600), anti-LC3B rabbit (1:400), and with the following secondary antibodies coupled with fluorescent protein: anti-rabbit Alexa Fluor 488 (1:300) and anti-mouse Cy3 (1:300). Confocal images were obtained with Olympus Fluorescent Confocal microscopy. White arrows indicate colocalizing vesicles. Scale bar = 5 µm. (**B**) Quantitation of percentage of merged LRP1/LC3 vesicles per cell with ImageJ Colocalization Finder software. (**C**) K562 cell lysates were subjected to WB. For protein detection specific primary antibodies were incubated and then secondary antibodies containing HRP were also incubated. *β*-actin were used as the loading control. (**D**,**E**) Quantitations of relative optic density (ROD) were obtained using ImageJ software. Data represent mean ± S.E.M. of three independent experiments. For immunofluorescence, 40 cells for each experiment were analyzed. Pearson values were compared statistically. *t*test and Tukey’s test for kinetic time comparison were performed. The significance of the *p*-values corresponds to *p*<0.05 (*), *p*<0.01 (**), and *p*<0.001 (***).

To determine the time at which LRP1 reached the autophagosomes, we performed a time-dependent kinetic stimulation with hemin and carried out an IF (Figure 3A). K562 cells were incubated for 1 h and also up to 24 h with hemin and compared with non-stimulated cells (Ctl). Afterward, cells were incubated with anti-LRP1β and LC3 antibodies to detect any endogenous proteins. A confocal analysis revealed a notable increase in LC3 and LRP1 positive vesicles from 8 h and up to 24 h of hemin stimulation (Figure 3A). A quantitation of the amount of LRP1 and LC3 positive vesicles showed that a constant increase had taken place in the colocalization of both markers in a time-dependent manner (Figure 3B).

Next, we examined whether LC3 and LRP1 also had a time-dependent kinetic response using immunoblot. To carry this out, we performed a WB in a lysate of K562 cells incubated with hemin for different times. It can be observed in Figure 3C,D that there was an increased level of LC3 II at 8 h of hemin stimulation, which still remained constant up to 24 h. Regarding LRP1, there was a notable K562 cell response to hemin stimulation after 1 h, increasing the amount of the receptor. However, no significant changes for higher stimulation times were noted, so LRP1 is not stimulated by hemin in a time-dependent manner (Figure 3E). WB also revealed that from 16 h of hemin stimulation a double-band of LRP1 appeared near to the 65 kDa molecular weight (Figure 3C). It can also be observed in the same figure that this double band did not appear in control cells. Taken together, these results indicate that hemin induced a time-dependent kinetic response of LC3 levels in K562 cells, with the peak of colocalization between LRP1 and LC3 at 8 h after the stimulation.

### Hemin induces autophagy through LRP1

**Figure 4:**
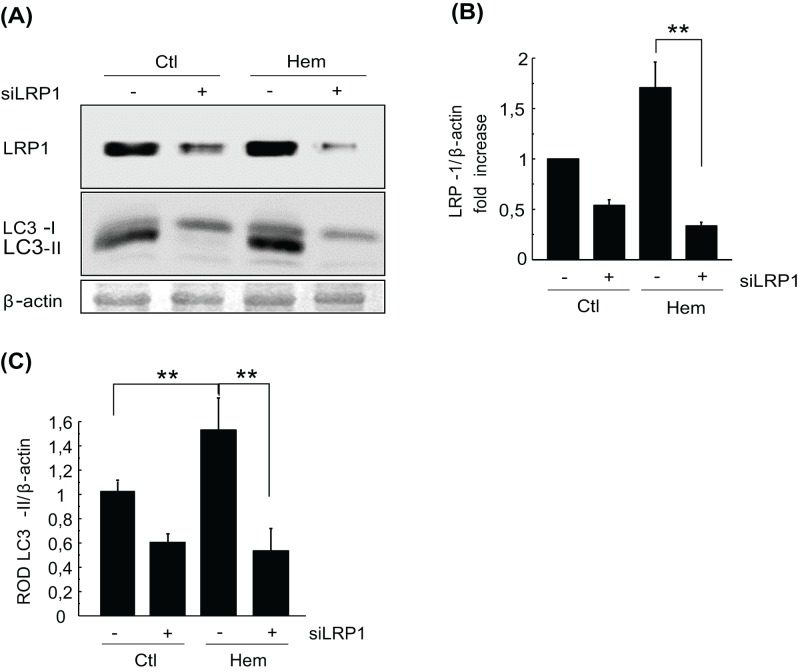
Hemin induces autophagy through LRP1 K562 cells were transfected with scramble plasmid (−) and siRNA (+) against LRP1 by double-hit electroporation. Then, cells were incubated in the absence (Ctl) or presence (hem) of hemin [50 µM] for 8 h. (**A**) WB of cell lysate and detection of specific protein with primary antibodies followed by secondary antibodies containing HRP. *β*-actin was used as the loading control. (**B**,**C**) Quantitation of relative optic density (ROD). Data represent mean ± S.E.M. of three independent experiments. For immunofluorescence, 40 cells for each experiment were analyzed. Pearson values were compared statistically. Statistical *t*test and Dunnett’s test for group comparison with control were performed. The significance of the *p*-values corresponds to *p*<0.01 (**).

As we have previously shown that hemin is able to induce autophagy and LRP1 overexpression, we were interested in determining whether LRP1 is involved in hemin-stimulated autophagy. For this purpose, K562 cells were first co-transfected with a GFP-vector plasmid and a scrambled siRNA against LRP1 and then incubated in the presence (Hem) or absence (Ctl) of hemin, with the LRP1 and LC3 protein levels being detected by WB (Figure 4A). A specific reduction occurred in the amount of the receptor, as shown in Figure 4B. In order to analyze if this knockdown of LRP1 affected the autophagy induction, the levels of LC3-II were analyzed. Interestingly, the levels of LC3 II were only reduced in cells transfected with LRP1 siRNA (Figure 4C), even for cells incubated in the presence of hemin (Figure 4C). These results suggest that LRP1 is required for autophagy induced by hemin.

Because LRP1 has an important role in autophagy stimulation, we wanted to see if overexpression of LRP1 was capable of promoting autophagy in the absence of hemin. Thus, K562 cells were transiently transfected with a GFP-vector or an expression vector codifying for a mini-receptor version of LRP1, known as mLRP4-GFP-HA. This mini-receptor contains the fourth ligand-binding domain of LRP1 and is capable of binding several LRP1 ligands with endocytic properties identical with full-length LRP1 (Obermoeller-McCormick L.M., 2001). A population of GFP-vector transfected cells were incubated in the presence of hemin as a positive control of stimulation, with WB analysis (Supplementary Figure S1A,B) and a confocal microscopy analysis (Supplementary Figure S1C,D) revealing that overexpression of mLRP4-GFP-HA was not able to induce autophagy.

In order to determine if the hemin effect was specific to the K562 cell line, we evaluated the role of the erythroid maturation inductor in another erythroblastic cell line (HEL 92.1.7 cells) and also in a non-hematopoietic cell line (HeLa cells). As shown in Supplementary Figure S2A,B, hemin induced a rise in LC3 lipidation in HEL 92.1.7 cells. This result was corroborated by IF microscopy to detect endogenous LC3 protein (Supplementary Figure S2D), with image quantitation showing that in hemin-stimulated cells there was a significant increase in the number of LC3 positive dots compared with control cells (Supplementary Figure S2E). Moreover, we observed a marked increase in the LRP1 level in HEL 92.1.7 cells incubated with hemin for 24 h (Supplementary Figure S2C). Similarly, hemin induced an increased level of the lipidated form of LC3 in HeLa cells (Supplementary Figure S3A,B), which was confirmed by fluorescent microscopy of an HeLa cell overexpressing GFP-LC3 WT plasmid (Supplementary Figure S3C,D). Another important observation was that hemin did not induce a significant stimulation of LRP1 protein for different times, and that the 65 kDa band of LRP1 observed in K562 cells from 16 h did not appear in the HeLa cell line (Supplementary Figure S3E,F). These data show that despite hemin being able to induce autophagy in HeLa cells, it could not stimulate any increment of LRP1 in this cell line.

### Hemin decreases LRP1 localization in early endosomes and recycling vesicles

As mentioned in the introduction, once LRP1 binds a ligand, it is endocytosed in a clathrin-dependent manner and is incorporated into early endosomes, where it them releases the ligand, before being recycled back to the plasma membrane.

**Figure 5:**
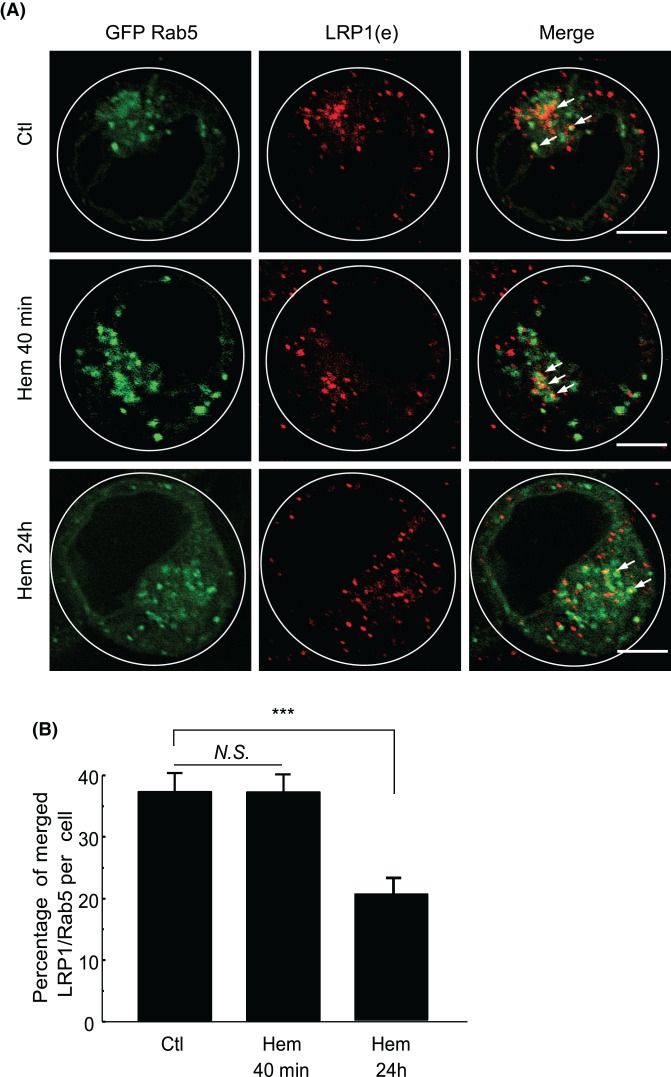
Hemin decreases LRP1 accumulation in early endosomes in K562 cells K562 cells were transfected with GFP-Rab5 wild-type (early endosomes). (**A**) Fluorescent confocal images of immunofluorescence of GFP-Rab5 K562 cells incubated for 40 min and 24 h with hemin [50 µM] (hem). Control cells were not stimulated (Ctl). Endogenous LRP1 were tagged with antibody coupled with Alexa Fluor 594. White arrows indicate colocalizing vesicles. Scale bar = 5 µm. (**B**) Quantitation of percentage of merged LRP1/Rab5 vesicles with ImageJ colocalization finder software. Data represent mean ± S.E.M. of three independent experiments. For immunofluorescence, a total of 40 cells for each experiment were analyzed. Pearson values were compared statistically, and a *t* test was performed. The significance of the *p*-values corresponds to *p*<0.001 (***).

To analyze whether hemin could affect the LRP1 trafficking in K562 cells, we investigated the localization of endogenous LRP1 at different times of hemin incubation by immunostaining some of the endocytic compartments. K562 cells overexpressing GFP-Rab5 (an early endosome marker) were incubated in the presence (Hem) or absence (Ctl) of hemin for 40 min (to observe the LRP1 localization at an early time of stimulation) or 24 h. Then, the LRP1 structures were detected using a specific antibody and subsequently analyzed by confocal microscopy (Figure 5A). Our results revealed that there were no differences in the number of Rab5 positive structures labeled with LRP1 in cells incubated in full nutrient or cells incubated with hemin for 40 min (Figure 5B), suggesting that LRP1 did not modify its localization in the early endosomes, even at an early time of hemin stimulation. In contrast, when cells were incubated with hemin for 24 h, there was a decrease observed in the percentage of colocalization between both markers, of ∼35% in the non-stimulated condition and approximately 20% in cells incubated in the presence of hemin (Figure 5B). This result suggests that hemin might trigger a possible mobilization of a population of LRP1 from the early endosomes.

**Figure 6:**
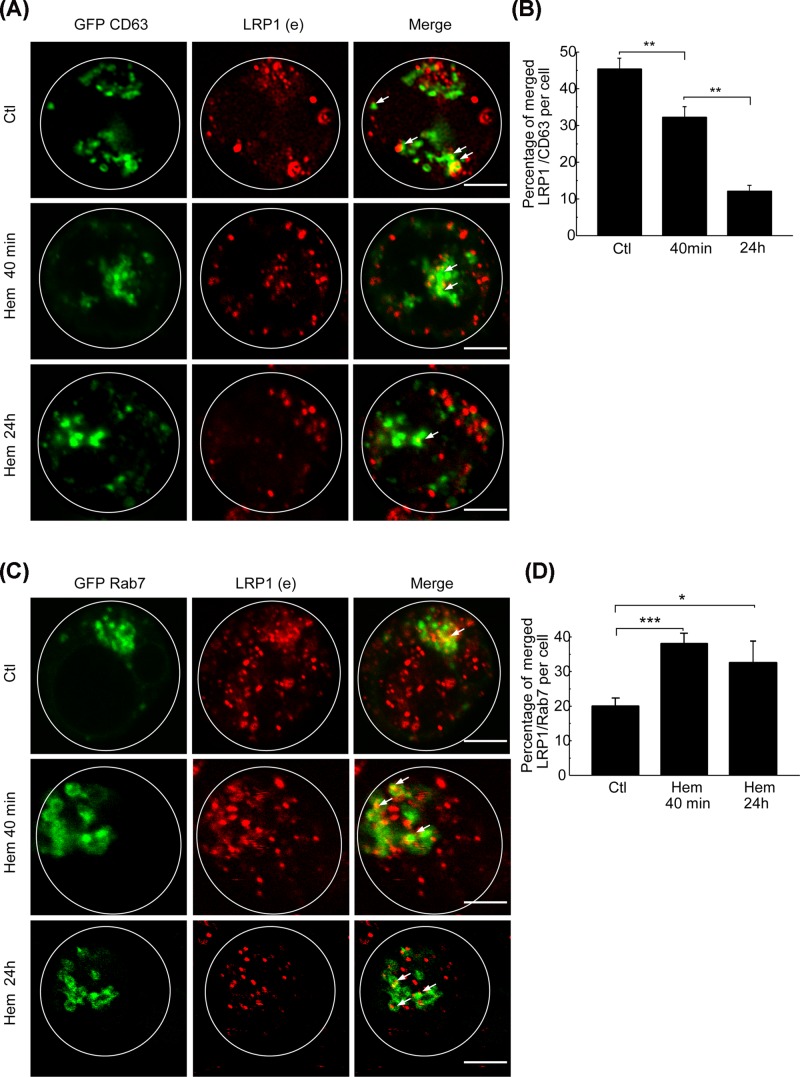
Hemin promotes LRP1 traffic modification in MVB in K562 cells (**A**) Fluorescent confocal images of immunofluorescence of GFP-CD63 in K562 cells incubated for 40 min and 24 h with hemin [50 µM] (hem). Control cells were not stimulated (Ctl). Endogenous LRP1 were tagged with antibody coupled with Alexa Fluor 594. Scale bar = 5 µm. (**B**) Quantitation of percentage of merged LRP1/CD63 vesicles with ImageJ colocalization finder software. (**C**) Fluorescent confocal images of immunofluorescence of GFP-Rab7 in K562 transfected cells incubated for 40 min and 24 h with hemin [50 µM] (hem). Control cells have not been stimulated (Ctl). Endogenous LRP1 were tagged with antibody coupled with Alexa Fluor 594. White arrows indicate colocalizing vesicles. Scale bar = 5 µm. (**D**) Quantitation of percentage of merged LRP1/Rab7 vesicles with ImageJ colocalization finder software. Data represent mean ± S.E.M. of three independent experiments. For immunofluorescence a total of 40 cells for each experiment were analyzed. Pearson values were compared statistically, and a *t* test were performed. The significance of the *p*-values corresponds to *p*<0.05 (*), *p*<0.01 (**), and *p*<0.001 (***).

Hemin is able to stimulate well-known recycling vesicles in K562 called MVBs [32,33], which are responsible for the accumulation and sorting of numerous membrane receptors. To address whether LRP1 is accumulated in MVBs, the K562 cells were transfected with a plasmid encoding GFP-CD63 (an MVB marker) and then incubated for 40 min or 24 h. These cells presented a high level of colocalization between LRP1 and GFP-CD63 in control conditions, indicating that LRP1 was accumulated in MVBs in basal conditions (Figure 6A, upper panel). Moreover, quantitation of the amount of LRP1 colocalizing with MVBs revealed that hemin produced a marked decrease in the amount of LRP1 and GFP-CD63 merged vesicles after 40 min (31%) and 24 h (10.5%) compared with control conditions (45%) (Figure 6B). These results indicate that in control conditions a population of LRP1 was localized in the MVBs. When, hemin was not stimulating the accumulation of LRP1 into MVBs, there existed a mobilization to other compartments with the longer stimulation time.

### Hemin causes relocation of LRP1 from late endosomes and autophagosomes to lysosomes

**Figure 7:**
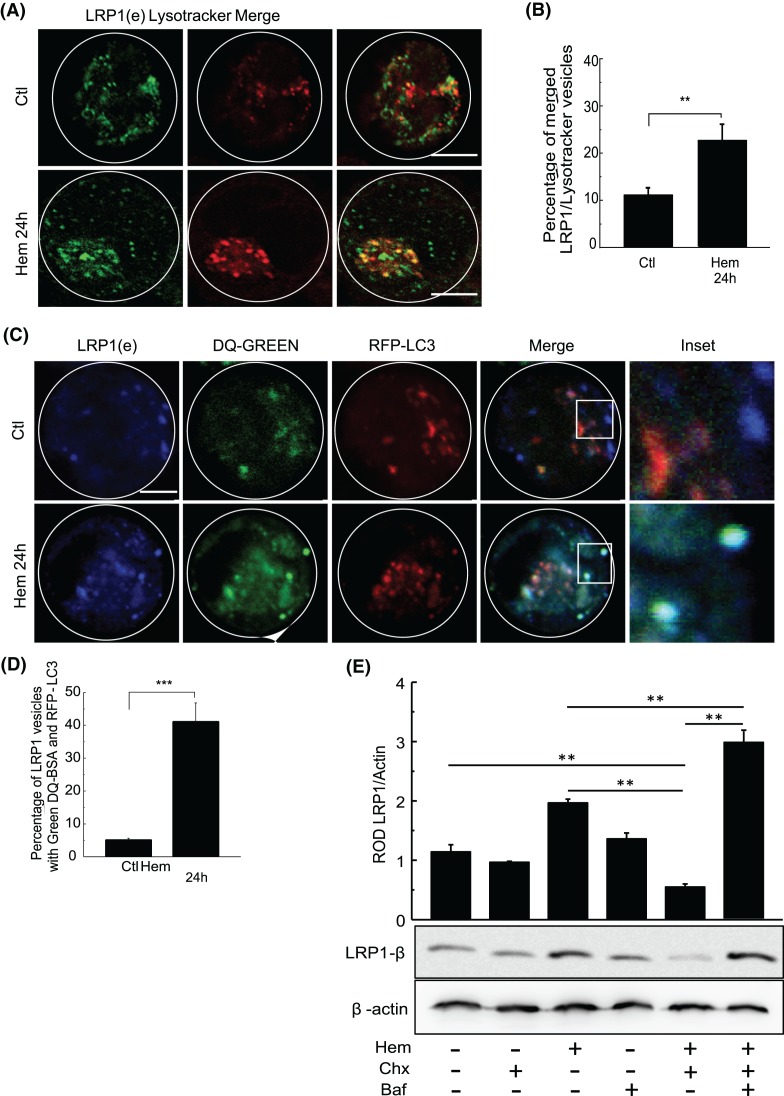
Hemin increases LRP1 accumulation in later endosomes and lysosomes in K562 cells (**A**) Fluorescent confocal images of immunofluorescence in K562 cells incubated for 24 h with hemin [50 µM] (hem). Control cells were not stimulated (Ctl). Lysotracker Red was added 30 min before stimulation cut and endogenous LRP1 were tagged with antibody coupled with Alexa Fluor 488. Scale bar = 5 µm. (**B**) Quantitation of percentage of merged LRP1/Lysotracker vesicles with ImageJ colocalization finder software. (**C**) Fluorescent confocal images of immunofluorescence in RFP-LC3 transfected K562 cells. Cells were incubated with or without hemin [50 µM] for 24 h, and then DQ-Green was added for 2 h before incubation stop. Endogenous LRP1 were tagged with antibody coupled with Alexa Fluor 405. Scale bar = 5 µm. (**D**) Quantitation of triple colocalization in merged cells with ImageJ software. Data represent mean ± S.E.M. of three independent experiments. (**E**) WB of untreated (Ctl) and hemin-treated cells in the presence or absence of protein inhibitor 10 µM cycloheximide (Chx) and in combination of bafilomycin A1 (Baf) for 4 h. For LRP1 protein detection, specific primary antibodies were incubated and then secondary antibodies containing HRP were incubated. *β*-actin was used as loading control. Quantitation of relative optic density (ROD) was obtained with ImageJ software. For immunofluorescence, 40 cells for each experiment were analyzed. Pearson values were compared statistically, and a *t*test were performed. The significance of the *p*-values corresponds to *p*<0.01 (**), and *p*<0.001 (***).

Following the endosomal pathway, we analyzed whether LRP1 was able to deliver to degradative compartments such as late endosomes (LE). K562 cells were first transfected with GFP-Rab7 wild-type plasmid, a well-known LE marker, and incubated in the absence (Ctl) or presence of hemin (hem) for 40 min and 24 h. This, cells were fixed and the endogenous LRP1β was immunolabeled (Figure 6C). The basal condition showed that LRP1 presented very little colocalization with Rab7 positive structures at either time (Figure 6C right panels). Interestingly quantitation of merged vesicles demonstrated that there was approximately a two-fold increase in the colocalization at 40 min and 24 h after hemin stimulation (Figure 6D). This percentage is in agreement with the approximately 20% reduction in LRP1 localized in Rab5 early endosomes. This result is consistent with the mobilization of LRP1 from early to late endosomes. Due to the receptor appearing to be associated with Rab7 vesicles, in K562 cells, we evaluated whether after hemin induction LRP1 could be targetted to degradative compartments. To carry this out, we performed IF of K562 cells without (Ctl) or with hemin (Hem) for 24 h. Next, Lysotracker Red was added for 30 min at 37°C, and the fixed cells were immunostained with anti-LRP1β antibody and evaluated by fluorescent confocal microscopy (Figure 7A). The quantitation of merged vesicles demonstrated that LRP1 had a very low localization in the degradative compartments in the control condition. In contrast, 24 h of hemin stimulation resulted in strongly significant increase in the colocalization of Lysotracker and endogenous LRP1 (Figure 7B).

To see if the results would be found using another specific lysosomal marker, we transfected K562 cells with RFP-Cathepsin D and performed analysis at 24 h of hemin induction (Supplementary Figure S4A). Quantitation of the merged vesicles showed, in line with previous results, that after 24 h of hemin incubation K562 cells presented an increase in the amount of LRP1 localized in the lysosomal structures (Supplementary Figure S4B). To examine whether this increased localization of LRP1 in autophagosomes also targetted LRP1 to degradative lysosomal structures we performed a triple immunostaining with LRP1, LC3, and DQ-green, with the later being a well-known marker to detect functional degradative lysosomes [34] (Figure 7C). A confocal analysis of merged images and quantitation demonstrated that in the control condition there was a very low triple colocalization, which is consistent with previous results. In contrast, when K562 cells were stimulated with hemin for 24 h, the triple localization showed a significant rise of more than 400% compared with the control condition (Figure 7D). These results confirm that hemin promoted a mobilization of LRP1 from endosomal vesicles to autophagolysosome structures. However, due to hemin inducing both LRP1 expression and localization of the receptor in degradative compartments, it is difficult to observe the degradation of LRP1 upon hemin treatment. To solve this issue, the level of LRP1 was analyzed by WB in K562 cells incubated in the absence (Ctl) or presence of hemin (hem), or in the presence of the eukaryote protein synthesis inhibitor cycloheximide (Chx), hemin plus cycloheximide (hem+Chx) or hemin plus cycloheximide and Bafilomycin A1 (hem+Chx+Baf) for 24 h (Figure 7E), with the ratio of LRP1/actin being quantitated. Consistent with the idea that hemin induces the degradation of LRP1 in lysosome structures, Figure 7E reveal that not only did cycloheximide inhibite the expression of LRP1 in cells incubated in presence of hemin (hem+Chx) but hemin was also able to reduce LRP1 levels with respect to the control (Ctl) or cycloheximide (Chx) conditions, with the protein synthesis being inhibited. In contrast, Bafilomycin A1 induced an increased level of LRP1 in the presence of hemin and cycloheximide, suggesting that the inhibition of the lysosomal activity induces LRP1 accumulation. Taken together, these results confirm that hemin induces the degradation of LRP1 in autophagic structures.

## Discussion

During erythropoiesis, the nucleated precursor undergoes several biochemical and morphological changes before reaching the final mature stage. One of these intracellular modifications is autophagy stimulation, which is necessary in order to complete the maturation process. However, it is not well understood how autophagy is induced throughout the erythroid maturation process. Previous publications from our laboratory have shown that different physiological and pharmacological erythroid maturation stimulators are able to induce autophagy in different human hematopoietic cell types, such as K562 cells, with hemin (a physiological compound that stimulates erythroid maturation) being a potent autophagy inductor [14]. Interestingly, it has been shown that hemin is capable of binding to the CD91/LRP1 protein, thereby inducing its internalization via endocytosis in several cell types [27,35–37]. In addition, other authors have shown that K562 cells has this receptor at the plasma membrane [38], suggesting that LRP1 could be implicated in the internalization of the hemin in this cell line.

In the present study, we have shown that LRP1 is necessary to induce the hemin-stimulated autophagy in human erythroid-like cells such as K562 and HEL 92.1.7 cell line. Consistent with our results, Dr Hirayama’s lab has demonstrated that LRP1 mediates vacuolating cytotoxin (VacA)-induced autophagy. Furthermore, knockdown of LRP1 inhibits generation of LC3-II in response to VacA [39]. Likewise, a very recent report has shown that lactoferrin from bovine milk promotes autophagy via AMPK activation through the LRP1 signaling pathway [40]. In agreement with these observations, an image analysis of K562 and HEL 92.1.7 cells incubated with hemin revealed both an increase in the size and the number of LRP1-contained autophagic vesicles. In contrast, these parameters were not modified by incubation of K562 cells with EPO, indicating the hemin specificity for inducing LRP1 accumulation in autophagic structures. However, despite the fact that hemin was able to induce autophagy in non-hematopoietic cell lines such as HeLa (cervical cancer cells), it did not induce any changes in the LRP1 levels. This implies that the induction effect of hemin over LRP1 is specific to erythroid cell lines.

Autophagy induction was confirmed for a time-course assay using fluorescence microscopy, which indicated that the accumulation of LRP1-labeled autophagosomes had a peak at 8 h of hemin incubation. Similar results were also obtained by WB, where LC3 processing revealed a maximum level at 8 h of hemin incubation. Remarkably, the only overexpression of LRP1 (mLRP4) did not induce an autophagy response in K562 cells, indicating the important role of the LRP1–hemin complex formation in to trigger this process. In contrast, silencing LRP1 abrogated the hemin-stimulated autophagy response. These results confirm that this receptor has a crucial role in hemin-induced autophagy in this cellular model. In addition, we have determined by real-time RT-PCR and WB that hemin generates an increased expression level of both, the LRP1 gene and protein.

Interestingly, other recent results have demonstrated that expression of mRNAseq of LRP1 is higher in erythroid precursors than in mature erythrocyte, with the receptor level having a peak at a later erythroid differentiation stage, which might be correlated with mitochondria removal stage by autophagy (mitophagy) [41,42]. Moreover, it has been shown at the same stage that LRP1 undergoes a transitional increment, and it is possible to observe an increased expression of the hemopexin level, which is correlated with the hemoglobinization stage [42]. These findings are in agreements with results showing that dendritic cells (DC) express LRP1 and that DC treatment with human defensins up-regulates the surface expression of LRP1 as well as the activation/maturation markers on these cells [43]. Similarly, some studies have indicated that Rosiglitazone, a well-known compound with antidiabetic properties, modulates LRP1 expression in a hepatocellular carcinoma cell line [42,44,45]. Consistent with these results, several investigations have demonstrated that LRP1 modulates the proliferation, survival and differentiation of numerous cell types such as neural stem cells and progenitors, adipocytes, Schwann cells, and vascular smooth muscle cells [46,47]. These data show that LRP1 has a crucial role in different cellular maturation processes and provide solid information on the possible role of LRP1 in regulating erythropoiesis. This receptor may be modulating the internalization of hemin to promote hemoglobin synthesis, with LRP1 being involved in the regulation of the mitochondrial iron exchanger receptor FLVCR1 [29], and also the degradation of mitochondria through hemin-induced autophagy [14].

Several reports have proposed that once the receptor is internalized, LRP1 is accumulated in early endosomes and releases the ligand to later compartments. LRP1 commonly colocalizes with the early endosomal marker EEA1, where the recycling pathway is then continued to the plasma membrane [20,24,25,48–50]. However, to date it is not completely understood how the cell modulates the intracellular LRP1 protein levels is not completed understood. Melman [51] proposed that LRP1 cellular turnover is regulated by the ubiquitin–proteasome system. On the other hand, it has been demonstrated that insulin induces a significant decrease in the LRP1 level in macrophages, which is mediated by the activation of the proteasomal system [52]. In contrast, some works have demonstrated that inhibition of the proteasomal system does not prevent complete LRP1 degradation [53,54], which suggests to us that there could be an alternative LRP1 degradation pathway. Interestingly, we have demonstrated herein that hemin caused a mobilization of LRP1 from the early and recycling endosomes to the autophagolysosomes, by its degradation. This agrees with the fact that LRP1 levels are practically undetectable in mature red blood cells, unlike their immature progenitors, suggesting that hemin may regulate the receptor level during the erythroid maturation process. In agreement with this observation, a recent report has shown in neurones that LRP1 colocalizes with the late endosomal marker Rab7, thus facilitating the transport of the *β*-secretase (BACE1) to the lysosomes for degradation [55]. Similarly, it has been reported that LRP1 undergoes lysosomal degradation in HepG2 cells incubated in the presence of Rosiglitazone [53]. Interestingly, Dr Joachim Herz’s laboratory has shown that the cytoplasmic domain of LRP1 is released by an enzyme with γ secretase activity [56]. In this context, we speculate that this secretase is probably responsible for the double-band of LRP1 obtained by WB after hemin stimulation.

Taken together, the results of our present study demonstrate, for the first time, that the physiological erythroid maturation stimulator hemin is able to induce autophagy in an LRP1-dependent manner. Moreover, we have presented evidence that hemin produces an accumulation of LRP1 in autophagic structures, which mobilizes the receptor from early endosomes to lysosomal compartments (autophagolysosomes). Interestingly, K562 cells have been used as a relevant hematopoietic proliferation and differentiation model [14,57–60], as well as a model to determine the possible therapeutic role of new compounds in hematological disorders. As autophagy induction is an indispensable event during the erythroid differentiation process, the mechanisms that promote autophagy during erythropoiesis steel be further explored. Related to this, our results suggest that hemin, via the LRP1 receptor, favors erythroid maturation by inducing an autophagic response in K562 cells, thereby making it a possible therapeutic candidate to help in the treatment of hematological disorders.

## Supporting information

**Supplementary Figure S1:** 

**Supplementary Figure S2:** 

**Supplementary Figure S3:** 

**Supplementary Figure S4:** 

## Abbreviations

DC: dendritic cell
EEA1: early endosome antigen 1
EPO: erythropoietin
FLVCR1: feline leukemia virus subgroup C
HA: hemagglutinin
LC3: light chain 3
LRP1: low-density lipoprotein receptor-related protein-1
VacA: vacuolating cytotoxin
WB: Western blot
