# Supplementary figures and images for: Hemin induces autophagy in a leukemic erythroblast cell line through the LRP1 receptor
Source: Biosci Rep. 2019 Jan 3;39(1):BSR20181156. doi: 10.1042/BSR20181156 (PMC6328880; doi:10.1042/BSR20181156)

Supplementary Figure 1

**A**

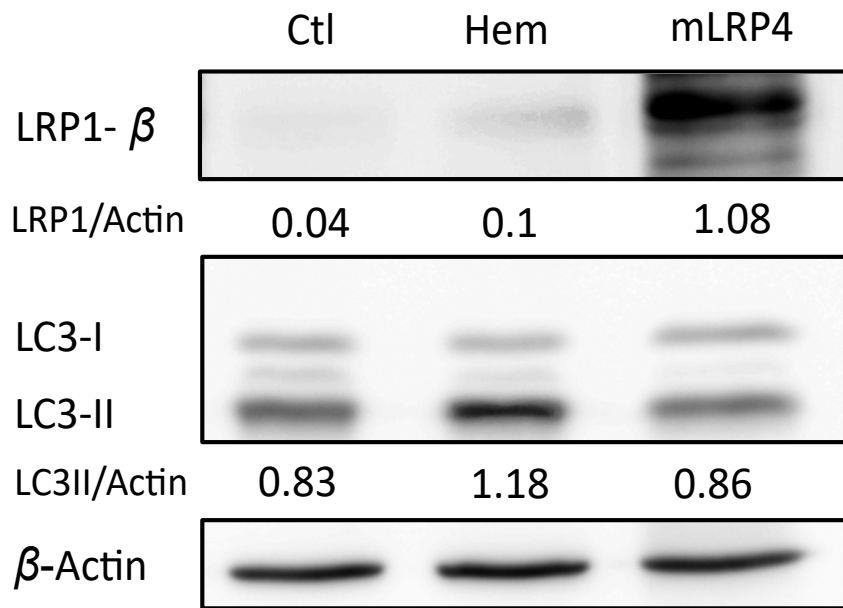

**B**

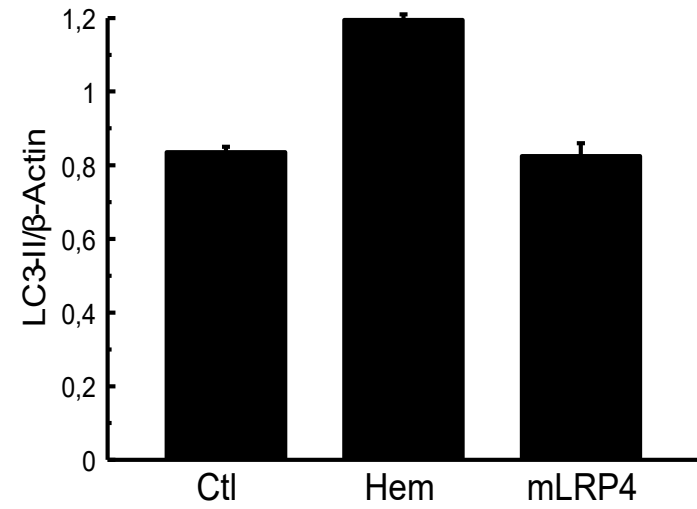

**C**

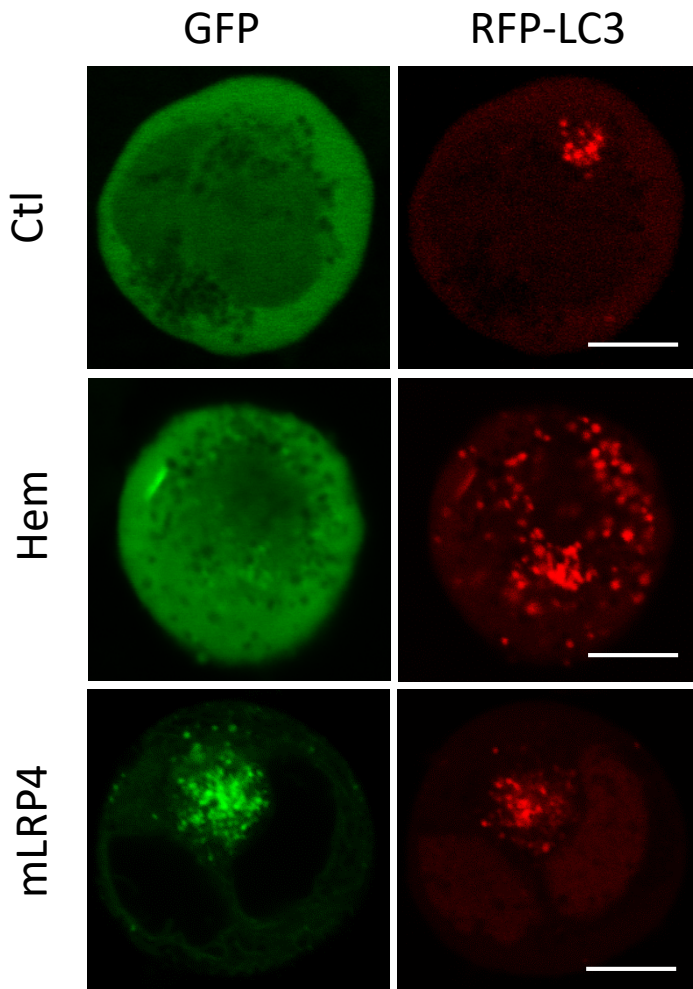

**D**

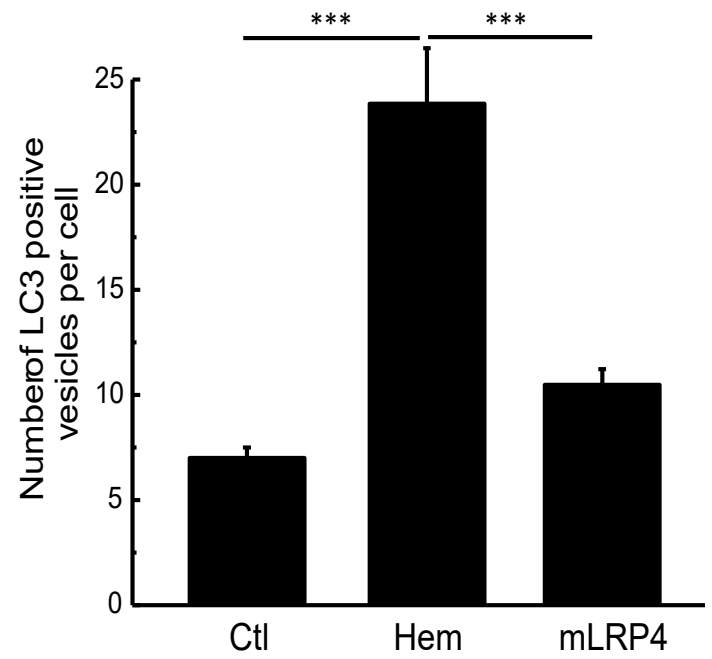

Supplementary Figure 2

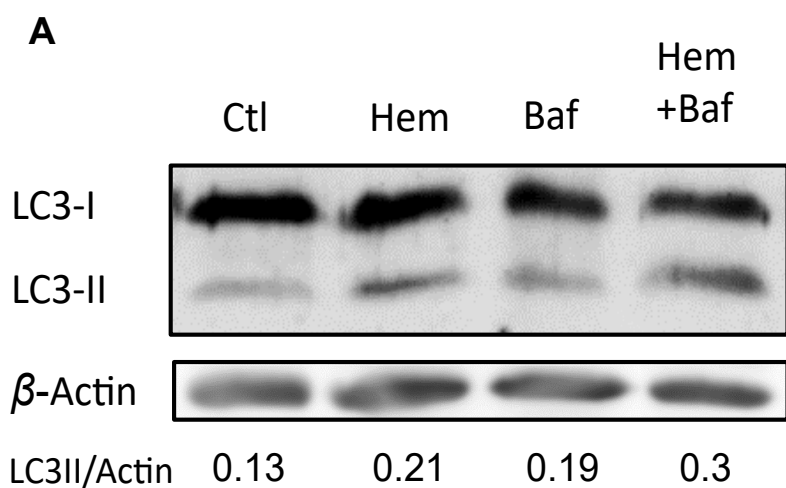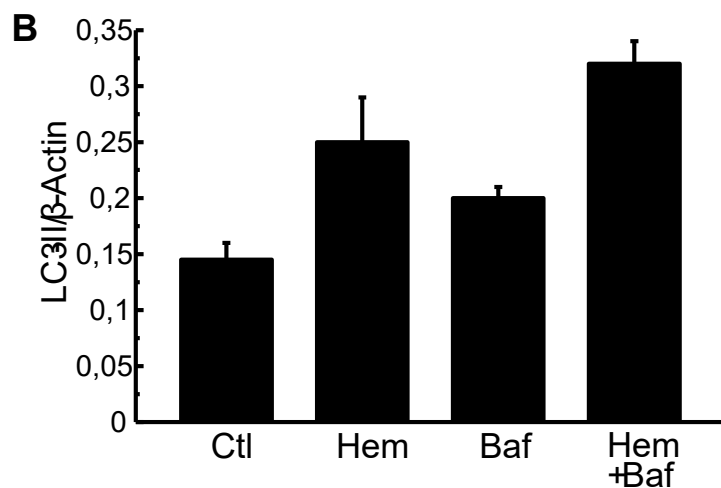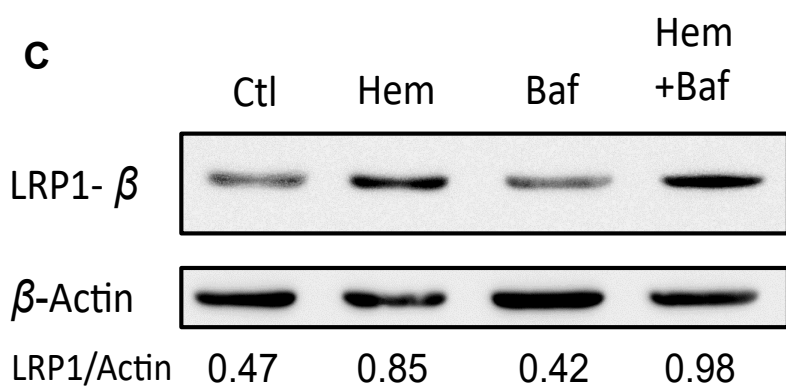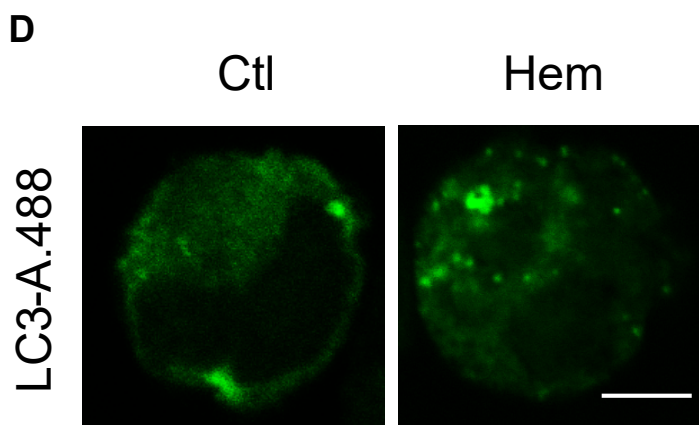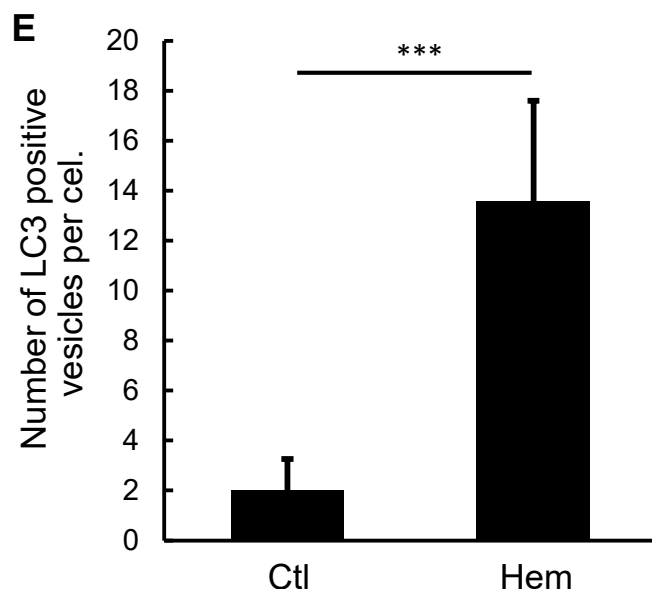

**A**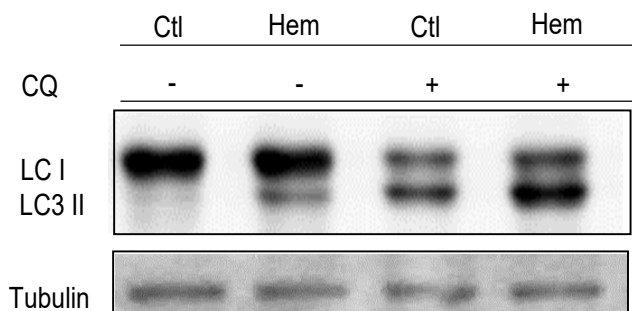**B**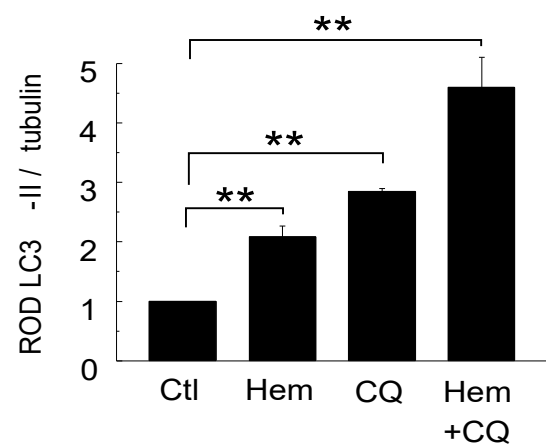**C**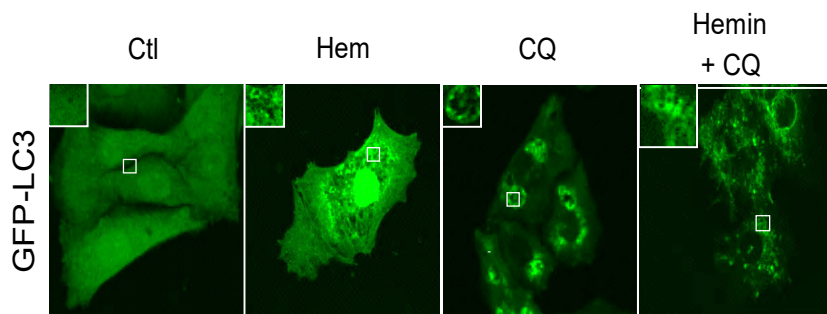**D**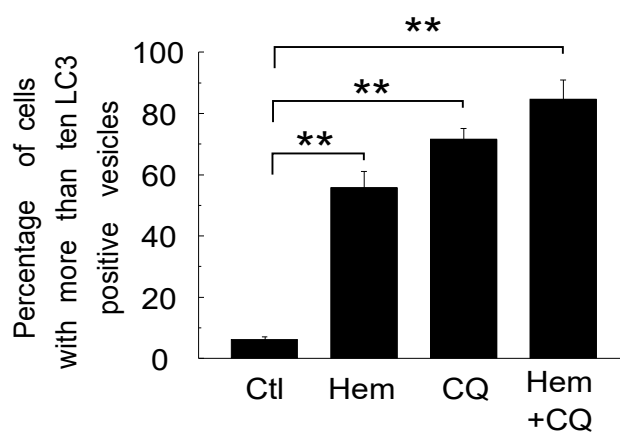**E**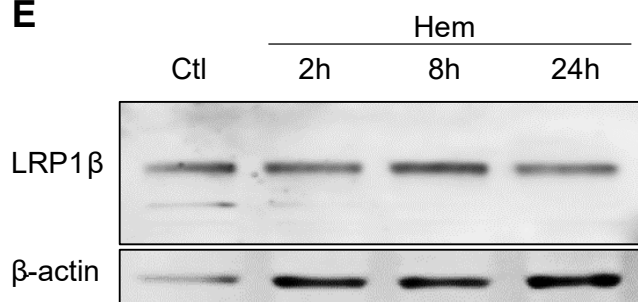**F**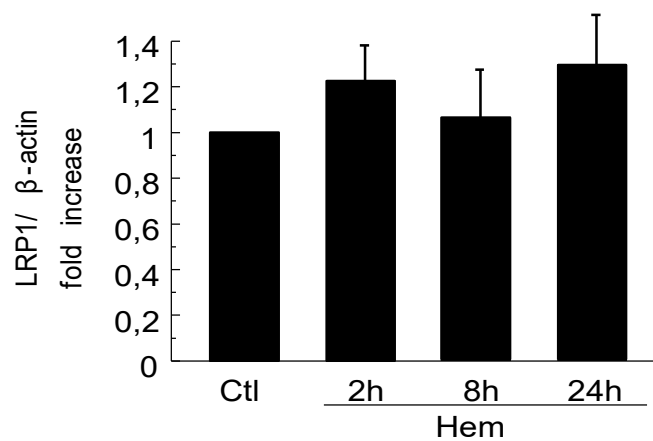

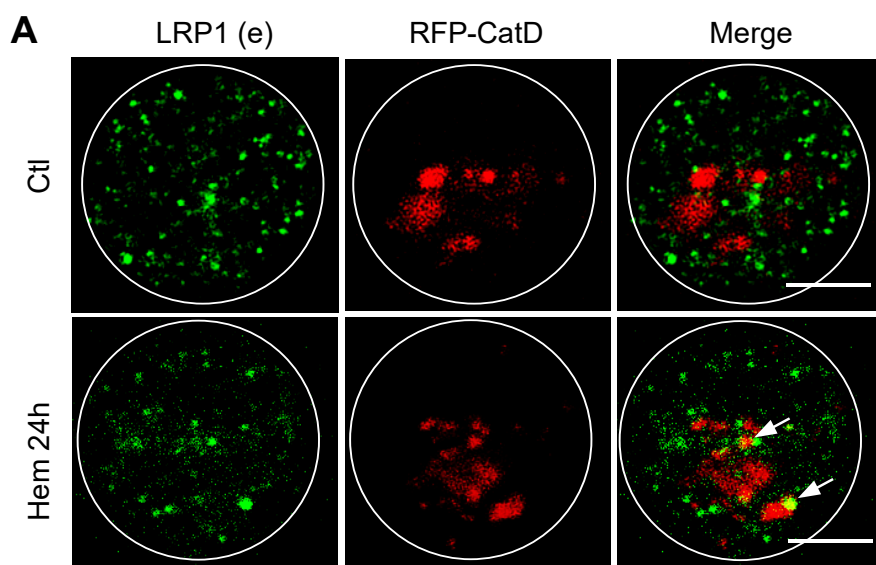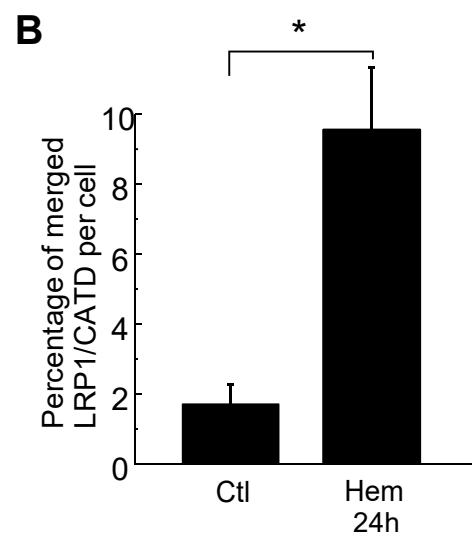

Supplement: Supplementary file 1 [file bsr20181156_Supp1.pdf]
